# Morphological assessment of cartilage and osteoarthritis in clinical practice and research: Intermediate-weighted fat-suppressed sequences and beyond

**DOI:** 10.1007/s00256-023-04343-2

**Published:** 2023-05-08

**Authors:** Patrick Omoumi, Charbel Mourad, Jean-Baptiste Ledoux, Tom Hilbert

**Affiliations:** 1 Department of Radiology, Lausanne University Hospital and University of Lausanne, Lausanne, Switzerland; 2 Department of Diagnostic and Interventional Radiology, Hôpital Libanais Geitaoui CHU, Achrafieh, Beyrouth, Lebanon; 3 Advanced Clinical Imaging Technology, Siemens Healthineers International AG, Lausanne, Switzerland; 4 LTS5, École Polytechnique FÉdÉrale de Lausanne (EPFL), Lausanne, Switzerland

**Keywords:** Osteoarthritis, MRI, MRI physics, Cartilage, Qualitative assessment, Artificial intelligence

## Abstract

**Abstract:**

Magnetic resonance imaging (MRI) is widely regarded as the primary modality for the morphological assessment of cartilage and all other joint tissues involved in osteoarthritis. 2D fast spin echo fat-suppressed intermediate-weighted (FSE FS IW) sequences with a TE between 30 and 40ms have stood the test of time and are considered the cornerstone of MRI protocols for clinical practice and trials. These sequences offer a good balance between sensitivity and specificity and provide appropriate contrast and signal within the cartilage as well as between cartilage, articular fluid, and subchondral bone. Additionally, FS IW sequences enable the evaluation of menisci, ligaments, synovitis/effusion, and bone marrow edema-like signal changes. This review article provides a rationale for the use of FSE FS IW sequences in the morphological assessment of cartilage and osteoarthritis, along with a brief overview of other clinically available sequences for this indication. Additionally, the article highlights ongoing research efforts aimed at improving FSE FS IW sequences through 3D acquisitions with enhanced resolution, shortened examination times, and exploring the potential benefits of different magnetic field strengths. While most of the literature on cartilage imaging focuses on the knee, the concepts presented here are applicable to all joints.

**Key points:**

1. MRI is currently considered the modality of reference for a “whole-joint” morphological assessment of osteoarthritis.

2. Fat-suppressed intermediate-weighted sequences remain the keystone of MRI protocols for the assessment of cartilage morphology, as well as other structures involved in osteoarthritis.

3. Trends for further development in the field of cartilage and joint imaging include 3D FSE imaging, faster acquisition including AI-based acceleration, and synthetic imaging providing multi-contrast sequences.

## Introduction

MRI is the preferred imaging modality for the morphological assessment of cartilage, allowing the direct visualization of chondral tissue and detection of cartilage substance loss. It is also a well-established method for the analysis of all joint tissues involved in osteoarthritis (OA), including menisci, ligaments, tendons, subchondral bone marrow, and synovium.

This review article will present the morphological MRI sequences used in clinical practice and clinical trials for the evaluation of osteoarthritis. We will discuss the strengths and weaknesses of fast/turbo spin echo fat-suppressed intermediate-weighted sequences (FSE FS IW), which are commonly used in most institutions for the morphological imaging of joints. Additionally, we will briefly discuss other sequences available for joint imaging in routine practice. While the focus will be on the assessment of cartilage, we will briefly mention other structures involved in OA, whenever relevant. Furthermore, we will provide an overview of some novel sequences and reconstruction techniques that are still under development, which may be useful for the morphological evaluation of cartilage and osteoarthritis. A description of compositional MRI techniques, which are currently mostly used in the research setting to probe cartilage ultrastructure quantitatively and detect early cartilage alterations, are beyond the scope of this review paper. Interested readers can refer to relevant literature for further information [1–4].

A substantial proportion of the cartilage imaging literature focuses on the knee and so does this manuscript. However, the considerations presented apply to all joints, even though the imaging of thinner cartilage is generally more challenging.

## Morphological imaging of cartilage, and of the rest of articular structures

### MRI as the modality of reference for the morphological assessment of osteoarthritis

While conventional radiography is still considered the first-line imaging modality for the assessment of osteoarthritis in clinical practice, cross-sectional techniques are required for direct visualization of cartilage substance loss, as well as a thorough assessment of other tissues involved in osteoarthritis, which is a disease of the whole joint.

**Fig. 1:**
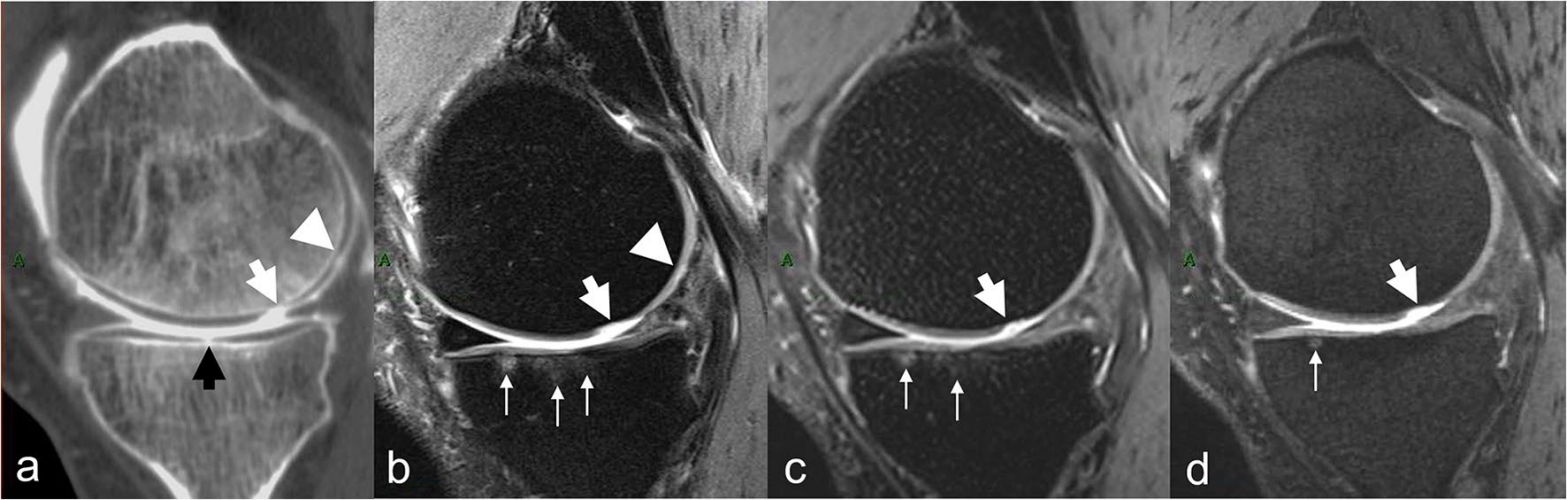
**a** Sagittal reformat of a CT arthrogram of the knee and corresponding **b** sagittal 2D fast spin-echo (FSE) fast-suppressed (FS) intermediate-weighted (IW), **c** sagittal reformat of 3D FSE IW FS and **d** sagittal reformat of 3D dual echo steady state (DESS) MR images obtained on a 3T MRI unit on the same day. Area of deep cartilage substance loss (arrow in **a**) is visualized on all MR images, whereas the superficial cartilage substance loss is only well depicted on 2D IW FS (arrowhead in **a** and **b**). Subchondral bone marrow edema-like signal intensity is visualized on 2D and partially on 3D IW FS (thin arrows in **b** and **c**) but much less conspicuous on 3D DESS image (thin arrow in **d**)

CT arthrography is an imaging modality of reference for the assessment of cartilage surface lesions, with several advantages: (1) it has higher spatial resolution than MRI, (2) the contrast between the low attenuation of cartilage and highly attenuating surrounding structures, i.e., overlying contrast-enhanced synovial fluid, and underlying subchondral bone, is higher than with MRI, and (3) it presents significantly shorter acquisition times [5] (Fig. 1). These factors contribute to higher diagnostic confidence for the study of cartilage. However, CT arthrography requires the intra-articular injection of contrast material, exposes patients to ionizing radiation, cannot detect intrasubstance lesions (lesions located in the cartilage substance and separated from the joint cavity by the remaining superficial cartilage layer), and only provides a partial assessment of other joint structures.

MR arthrography is another invasive technique, which for many joints, does not provide any added value compared to MRI for the assessment of osteoarthritis. For the knee joint, it has an added value in specific indications like the assessment of postoperative meniscus and of the stability of some chondral and osteochondral lesions [6–8].

For all these reasons, conventional MRI is widely considered the modality of reference for the morphological assessment of osteoarthritis.

### Intermediate-weighting demystified: the rationale behind using 2D FSE FS IW sequences

**Fig. 2:**
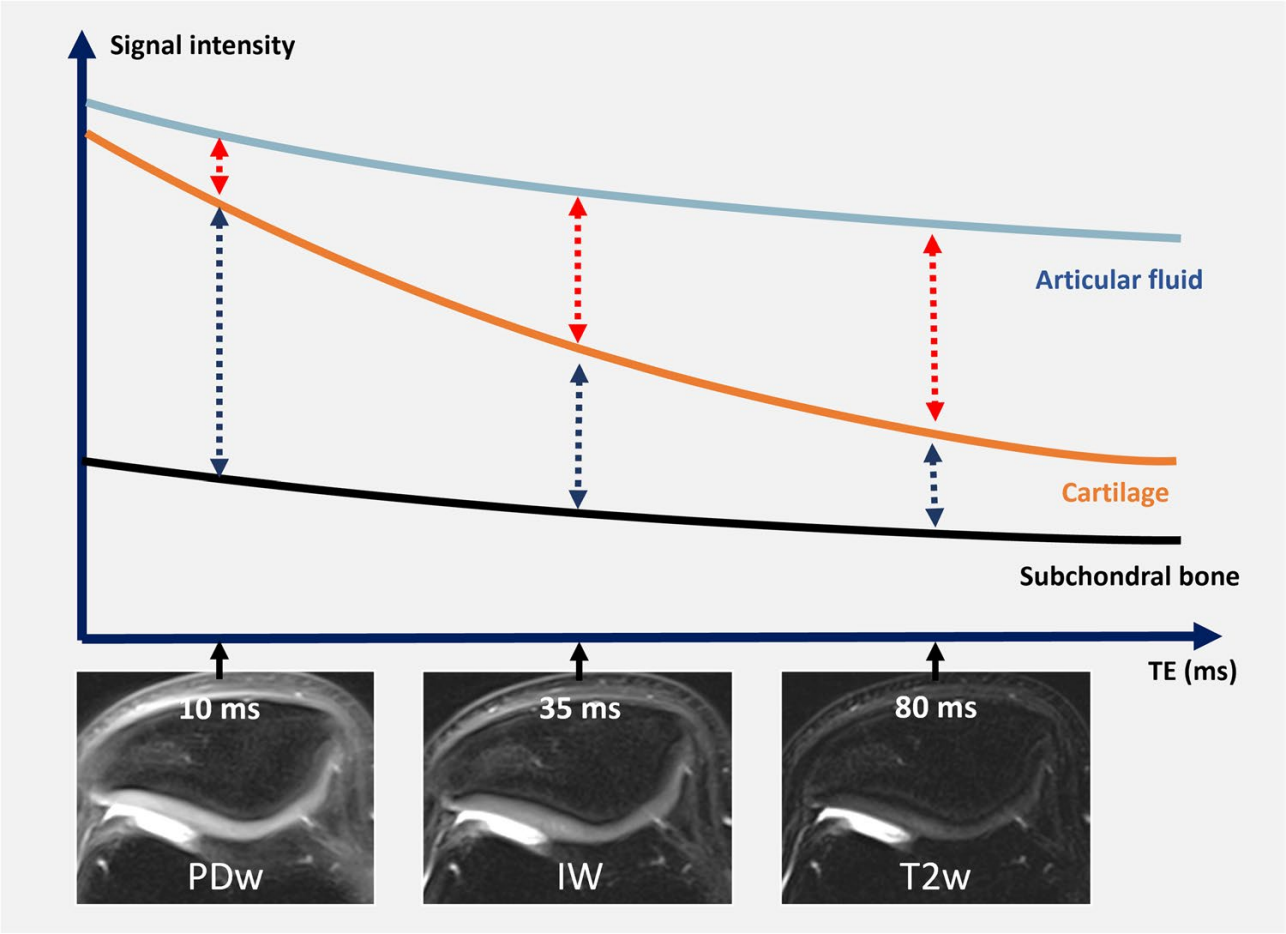
Schematic representation of the transverse magnetization decay of articular fluid, cartilage, and subchondral bone plate. Fat-suppressed proton density-weighted (TE=10ms), intermediate-weighted (TE= 30–40 ms), and T2-weighted (TE=80ms) transverse MR images of the patella of an asymptomatic 23-year-old male volunteer obtained on a 3T MRI unit. Intermediate-weighted images with a TE between 30 and 40 ms yield the best compromise in terms of intra-substance chondral signal and contrast, as well as of contrast between cartilage, synovial fluid, and subchondral bone

**Fig. 3:**
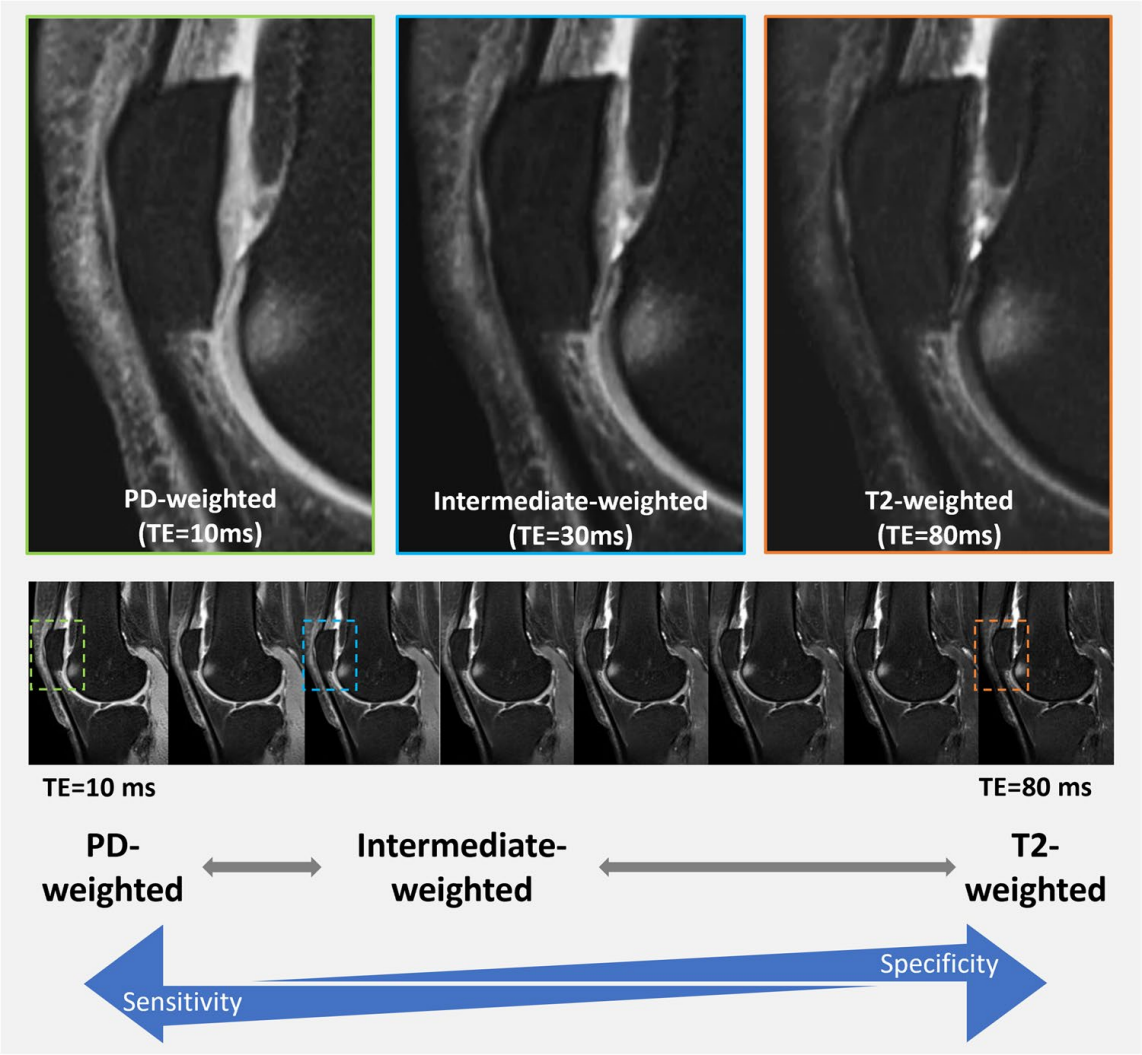
Sagittal fat-suppressed MR images with serial TEs ranging from 10 to 80ms (images from lower row) synthesized from a single multi-TE T2 map acquisition (GRAPPATINI ®) obtained on a 3T MRI unit, with selected zoomed in images (upper row) corresponding to PD-weighted, intermediate-weighted and T2-weighted. Images are windowed identically. Note that with increasing TE values, signal of lower T2 tissues, including cartilage, decays, but the contrast between fluid and cartilage increases. The higher the T2-weighting (i.e., the higher the TE), the lower the sensitivity, but the higher the specificity to fluid signal

**Fig. 4:**
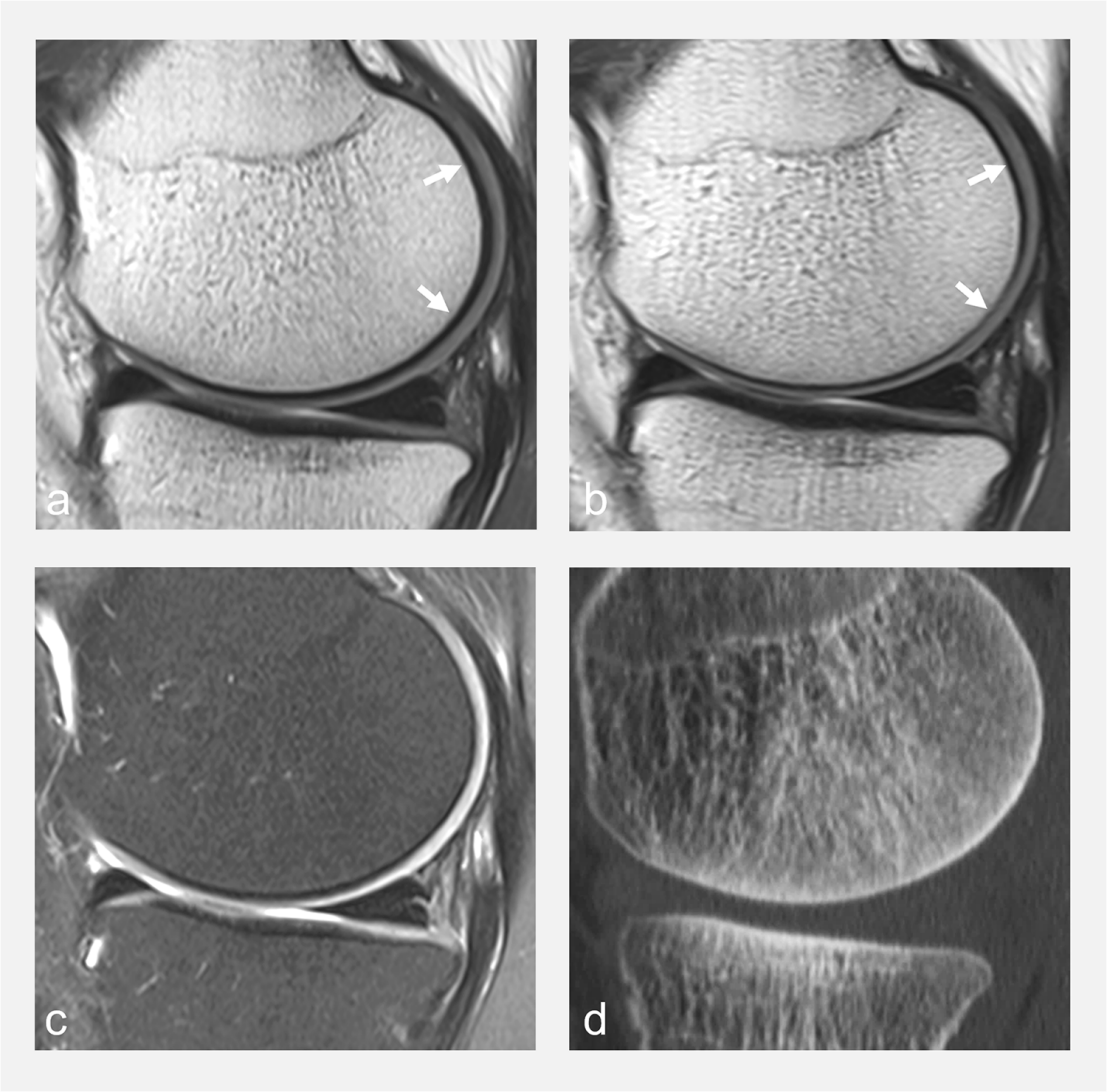
Sagittal non-fat-suppressed intermediate-weighted MR images obtained on a 3T MRI unit with different frequency-encoding directions in **a** anteroposterior, **b** craniocaudal, **c** fat-suppressed intermediate-weighted (anteroposterior frequency-encoding**),** and **d** sagittal CT reformat at the level of the medial femorotibial compartment, obtained the same day, in a 45-year-old asymptomatic volunteer. Note the variable appearance of the bone-cartilage interface (arrows in **a** and **b**) related to chemical shift artifact that occurs in the frequency encoding direction. The low signal intensity line at the interface should not be interpreted as the subchondral bone plate (compare with CT image **d** where the thickness of the subchondral bone plate can be appreciated). Fat suppression in **d** reduces chemical shift artifacts and increases contrast at the bone–cartilage interface

Among the variety of MRI sequences that exist, fast/turbo spin echo sequences (FSE) fat-suppressed (FS) intermediate-weighted (IW) sequences have been established over the last decades as the best compromise for the study of cartilage and joint structures [9–13]. FSE FS IW FS sequences are fluid-sensitive sequences with an echo time (TE) of 30 to 40ms, which is intermediate between proton-density-weighted (PDw) sequences (short TE around 10–15ms), and T2-weighted (T2w) sequences (long TE 70/80ms at 3T/1.5T respectively) (Fig. 2). Therefore, both the contrast and signal of IW sequences are intermediate between PDw and T2w sequences. IW sequences are a good compromise between the higher sensitivity of PDw sequences to water signal intensity and the higher specificity of T2w sequences (Fig. 3). Sequences with shorter TEs are more sensitive to visualize components with shorter T2 (both bound and free water) whereas sequences with longer TEs are more specific to free water. This implies that if a lesion is visible on a longer TE sequence, the lesion is likely associated with substance loss, and therefore filled with synovial fluid. These principles have been used in MRI of the meniscus, with shorter TE sequences being slightly more sensitive, and longer TE sequences more specific to the presence of a tear. A meniscal signal abnormality detected on short TE sequences is more likely to be a tear (filled with fluid) if the signal abnormality is also visible on T2w sequences [14, 15]. IW sequences provide good contrast between cartilage and adjoining structures (synovial fluid and subchondral bone). Additionally, FSE FS IW sequences are less sensitive to the magic angle effect than proton-density-weighted sequences, which is useful for the study of low T2 collagen-rich structures such as ligaments and menisci (Table 1) [16, 17]. The application of fat suppression provides additional benefits: (1) it increases the dynamic range of the image (by suppressing the signal of fat which has longer T2 values than the tissues of interest), and hereby increases contrast both inside the cartilage and between cartilage and surrounding structures, (2) it reduces chemical shift artifacts (Fig. 4), and (3) it enables the detection of bone marrow changes (Fig. 1) [10, 18].

**Table 1:** Strengths (bold) and drawbacks (italic) of commonly used MRI sequences for whole joint evaluation in knee osteoarthritis

	General	Cartilage	Bone marrow	Menisci	Osteophytes	Synovitis/effusion	Ligaments
**2D PDw FS** (TE 10–15ms)	**-Higher in-plane spatial resolution than 3D images** **-Shorter acquisition time** *-Anisotropic voxels* *-Section gaps* *-Partial volume effect*	**-Highest sensitivity to cartilage signal** *-Less contrast between cartilage and fluid* *-More prone to magic angle phenomenon*	**-Most sensitive**	**-Most sensitive**	*-Less sensitive than T1w*	**-Very sensitive to detect effusion** *-Less sensitive to detect synovitis than Fat suppressed T1w with IV contrast*	**-Most sensitive**
**2D T2w FS** (TE 70–80ms)		**-Highest contrast between cartilage and fluid** **-Least susceptible to magic angle phenomenon** *-Less contrast between deep cartilage and subchondral bone.* *-Less sensitive to intrinsic cartilage signal abnormalities*		**-Most specific**			**-Most specific**
**2D IW FS** (TE 30–40ms)		**-Best compromise for contrast and signal, and between sensitivity and specificity** **-Less magic angle phenomenon**		**-Best compromise between sensitivity and specificity**			**Best compromise between sensitivity and specificity**
**3D DESS**	**-Isotropic images with multiplanar capacity** **-Decreased partial volume effect** *-Lower in-plane spatial resolution than 2D images* *-Long acquisition time*	**-High SNR** **-High contrast between cartilage and fluid** *-Unreliable depiction of intrinsic cartilage signal intensity* *-Vulnerable to susceptibility artifacts*	*-Unreliable*	*-Unreliable*	**-More sensitive than 2D imaging**	**-Sensitive to detect effusion** *-Not sensitive to detect synovitis*	*-Unreliable*
**3D FSE IW FS**		**-High sensitivity to cartilage signal** **-Improved visualization of superficial cartilage defect** *-Prone to image blurring*	**-Equivalent to 2D FS images**	**-Equivalent to 2D FS images**		**-Very sensitive to detect effusion** *-Less sensitive to detect synovitis than Fat suppressed T1w with IV contrast*	**-Equivalent to 2D FS images**
**CT-arthrography**	**-Highest spatial resolution** **-Isotropic images with multiplanar capacity** **-Post-op setting (less prone to metallic artifacts)** **-Fast** *-Uses ionizing radiation* *-Necessitates intra-articular injection of contrast*	**-Highest sensitivity to evaluate lesions of cartilage surface (highest contrast between cartilage and surrounding structures)** *-Cannot detect intrasubstance lesions that do not communicate with articular surface*	*-Not sensitive* *-DECT is currently being assessed*	**-High sensitivity and specificity**	**-Standard of reference**	**-Aspiration and fluid analysis could be performed if necessary** *-Low sensitivity for synovitis (indirect assessment of synovial lining)*	*-Less sensitive*

*DESS* dual echo steady state, *IW FS* intermediate-weighted fat suppressed, *PDw* proton density-weighted, *T2w* T2-weighted

^*^For the sake of simplicity, other gradient-echo-based 3D images that are also used (SPGR, bSSFP, DEFT...) are not presented

Interestingly, FSE FS IW sequences were also found useful for a comprehensive whole joint assessment in OA, including bone marrow edema, synovitis/effusion, ligaments, and menisci [19, 20].

However, it is worth mentioning that there is great confusion on the terminology and definition of FSE FS IW in the literature, with reported TEs varying from 13 to 60ms [11, 21]. On the other hand, sequences with TE values between 30 and 40ms are often mistakenly referred to as PDw [22–25]. In practice, it is also important to note that some manufacturers implement FSE FS IW sequences as standard for their musculoskeletal applications, sometimes referring to them as “proton-density” sequences. Therefore, the readers are encouraged to check these specific sequence parameters.

### Standard MRI protocol for the morphological assessment of osteoarthritis: a combination of 2D sequences

The standard protocol for the assessment of osteoarthritis generally does not differ from the protocols used for the assessment of internal derangement of joints. Although these protocols vary greatly from institution to institution, they usually include one or more 2D FSE FS IW sequences in different planes, as well as T1-weighted sequences (useful for the assessment of bone in general, including osteophytes), and T2-weighted sequences, which are more specific to the signal of liquids (Fig. 3), less prone to the magic angle effect, and which may be useful for the characterization of meniscal, tendinous, and ligamentous tears. Although more specific, T2-weighted sequences are, however, less sensitive than FSE FS IW to pathological lesions, especially if not liquid-filled (Fig. 3).

In the research setting and for clinical trials, a combination of sequences that includes FSE FS IW sequences has been primarily recommended as part of the acquisition protocol for the assessment of osteoarthritis [26]. The last decade has seen the surge of 3D imaging, in particular spin-echo based sequences, which in some cases may replace the combination of 2D sequences (see below).

### Interpretation of morphological cartilage imaging with FSE FS IW sequences: pearls and pitfalls

FSE FS IW sequences display good intrinsic contrast within the cartilage substance as well as between cartilage and adjacent structures. However, a few interpretation pearls must be acknowledged, related to physiological variations in cartilage thickness from area to area, artefactual signal changes, as well as pathological signal changes.

#### Normal variations of cartilage thickness

**Fig. 5:**
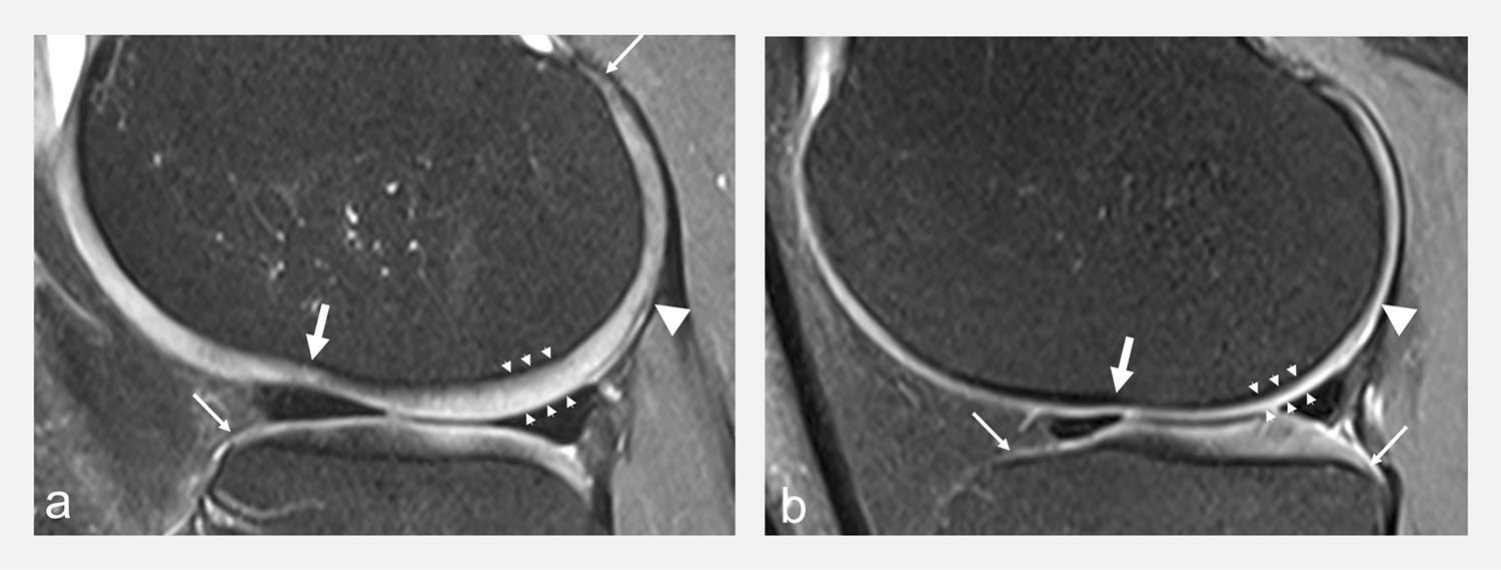
Sagittal FS IW MR images at the level of the lateral femoral condyle in **a** a 46-year-old asymptomatic male volunteer and **b** a 35-year-old asymptomatic female volunteer showing a normal inter-individual variation of cartilage thickness (between arrowheads), and physiological areas of cartilage thinning overlying the lateral femoral condylar notch (white arrows), the posterior lateral femoral condyle (arrowheads), and at the margins of cartilage (thin arrows)

Cartilage thickness has high interindividual variability and is greater in males [27, 28]. Previous reports on normal values of cartilage thickness and distribution have shown that cartilage thickness varies within joints. Notably, the lateral tibial cartilage is thicker than the medial, the medial femoral cartilage is thicker than the medial tibial cartilage while the opposite is true laterally, and the peripheral regions usually display thinner cartilage than central regions [28]. There are also areas of physiological cartilage thinning that frequently occur in specific anatomical areas such as the margins of cartilage, the lateral femoral condylar notch as well as the posterior aspect of the lateral femoral condyle [21, 29] (Fig. 5).

#### Artefactual variations of cartilage signal intensity

**Fig. 6:**
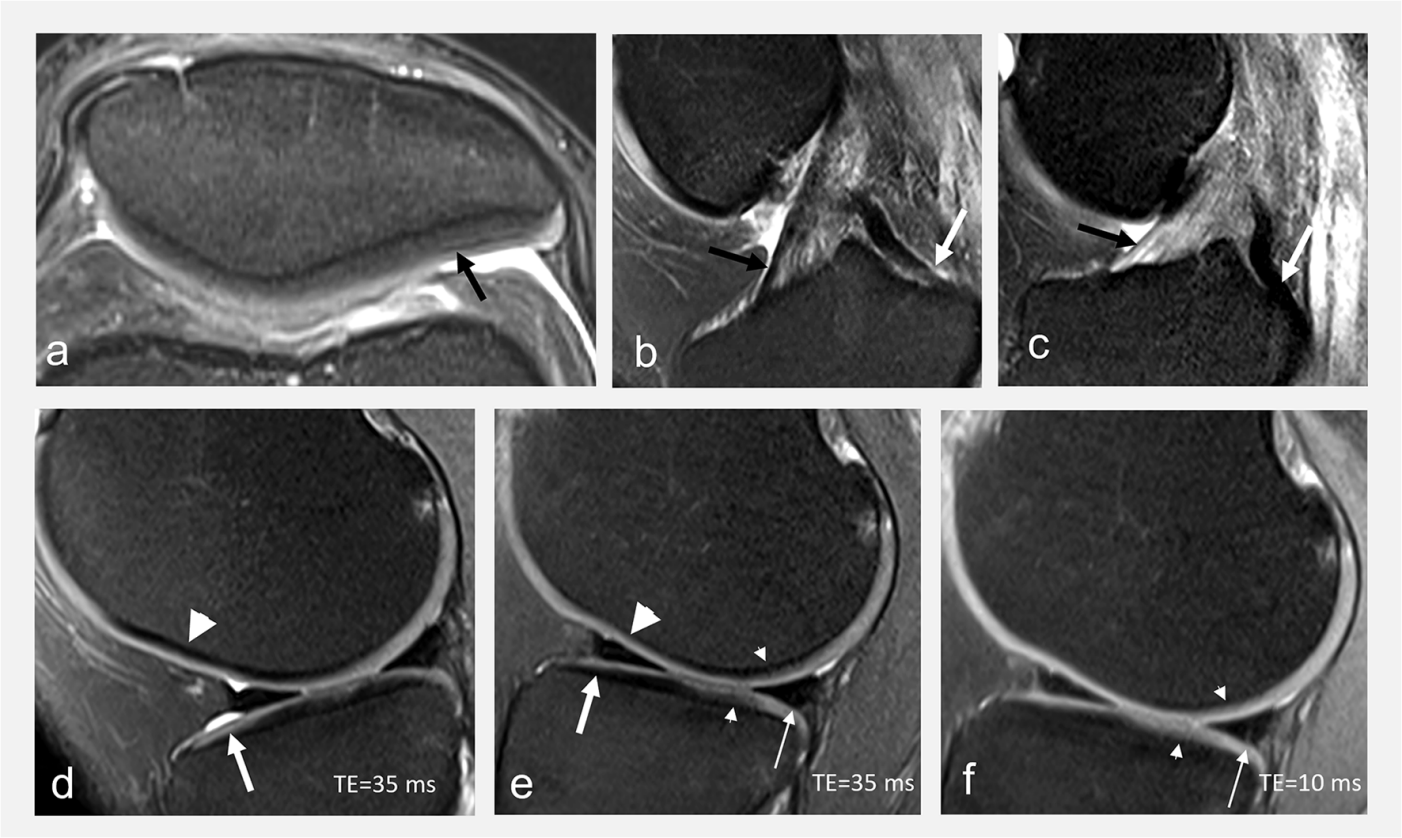
**a** Transverse FS IW MR image obtained on a 3T MRI unit showing truncation artifact (arrow in **a**) that gives a “false” laminar appearance to the patellar cartilage. Truncation artifacts typically appear as fine parallel lines immediately adjacent to high-contrast interfaces. Sagittal FS IW images in **b** flexion and **c** extension in a 36-year-old asymptomatic volunteer showing a variable signal intensity of the anterior (black arrows in **b** and **c**) and posterior (white arrows in **b** and **c**) cruciate ligaments related to the magic angle effect. Sagittal FS IW images in **d** flexion and **e** extension, and **f** sagittal PD fat-suppressed images in extension in the same volunteer obtained on a 3T MRI unit: the signal intensity of the cartilage (arrows) and the chondro-osseous junction (arrowheads) varies according to the orientation within the main magnetic field due to the magic angle effect. The signal intensity of cartilage (small arrows in **e** and **f**) also vary according to the TE. Note the fine meniscal high signal intensity line (**f**) that does not univocally reach the surface, seen on one image, and not visible on IW FS images (**e**), interpreted as no tear

Throughout the chondral tissue, from surface to depth and from region to region, normal variations of cartilage signal occur due to the fibrillar pattern of its ultrastructure, organized in layers defined by the collagen fibers orientation (superficial, transitional, and radial zones), and the magic angle effect [12, 22]. Chemical shift artifacts might alter the appearance of the chondro-osseous junction and hinder proper assessment of cartilage thickness, and a false laminar appearance of cartilage may occur due to truncation artifact [22, 30]. While a comprehensive review of MR artifacts is beyond the scope of this manuscript, Fig. 6 illustrates some common pitfalls in cartilage imaging.

#### Pathological variations of cartilage signal intensity

**Fig. 7:**
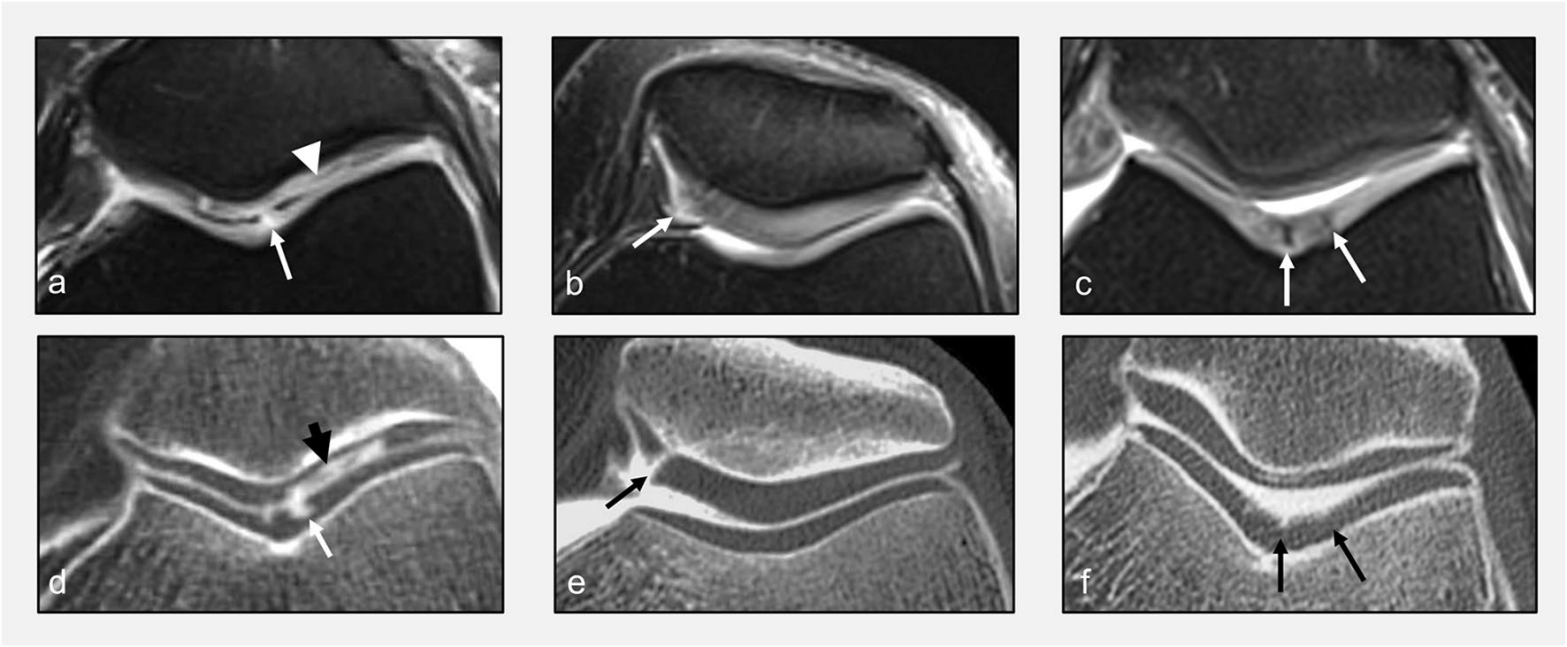
Transverse FS IW MR images at the level of the femoropatellar joint (**a**–**c**) and corresponding transverse reformats of CT arthrograms (**d**, **e**) obtained the same day. On MRI, cartilage fissures or substance loss can have variable signal intensity: hyperintense (arrow in **a**), isointense (arrowhead in **a**), heterogeneous (arrow in **b**), or hypointense (arrows in **c**)

Cartilage lesions more often translate into areas of high signal intensity. However, it should be acknowledged that cartilage lesions may also be hypointense, isointense, or present as an area of heterogeneous signal intensity, depending on the configuration of cartilage fibers (Fig. 7) [31–33].

In clinical practice, cartilage lesions are commonly graded based on their depth according to the Outerbridge or modified Noyes classifications [12, 34–36]. In addition, the maximum diameter of the lesion is a useful parameter for clinical decision-making [34, 37]. In the research setting, cartilage morphology is graded as part of semi-quantitative whole-organ scoring systems. A detailed description of these grading systems can be found in the literature [1, 38–40].

### Diagnostic performance of 2D morphological MRI of cartilage

There is currently no report focusing on the diagnostic performance of FSE FS IW sequences specifically. In a recent meta-analysis, the pooled sensitivity and specificity of 2D sequences acquired in multiple planes were 76 and 93%, respectively, for detecting cartilage lesions [41]. But a closer look at the papers analyzed in this report finds TE values covering the entire range of fluid-sensitive sequences from 13 to 119ms for 2D sequences. It should be noted that the diagnostic performance and interobserver agreement for the detection of cartilage lesions without substance loss (superficial fibrillation, or intrasubstance signal changes, corresponding to Outerbridge≤1 or modified Noyes≤2A lesions) is relatively low with standard morphological imaging, although the clinical significance of low-grade cartilage defects is uncertain [29, 42, 43]. Spatial resolution is a major determinant of diagnostic performance at imaging [44, 45]. Concerning delaminating cartilage lesions, to the best of our knowledge, the diagnostic performance of FSE FS IW sequences has only been assessed for the hip, with conflicting results [46–48]. Some authors advocate the use of traction MR arthrography to improve the diagnostic performance in this indication [49].

## Beyond 2D FSE FS IW sequences

While 2D FSE FS IW sequences are still widely used in clinical practice, other sequences have become available and are being used by some groups mostly in the research setting, either as a substitute of or in addition to FS IW sequences. The following paragraphs briefly describe the technical grounds of these sequences, citing their main strengths and limitations in relation to osteoarthritis imaging, considering both the clinical and research settings.

### Different types of fat suppression techniques

**Fig. 8:**
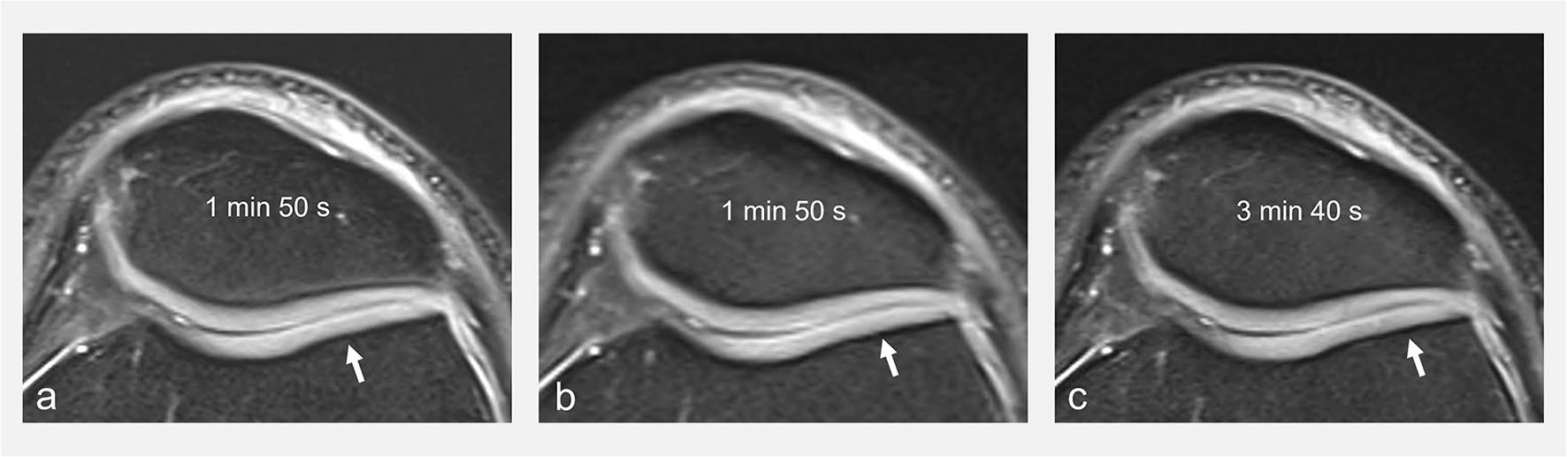
Transverse FS (CHESS) IW MR image (**a**), and corresponding **b**, **c** IW water Dixon MR images with different acquisition times (**b**, **c**) obtained on a 3T MRI unit. All other parameters kept equal, Dixon sequences are longer to acquire than CHESS fat-suppressed sequences (compare **a** and **c**). Note the better definition of the articular cartilage surface in **a** and **c** compared to **b** (arrows), due to higher spatial resolution

As discussed above, suppressing the signal of fat increases the dynamic range of the image and hereby the contrast in and between structures of interest. The most widespread fat suppression techniques in musculoskeletal imaging have been chemical shift selective (CHESS) and short-tau inversion recovery (STIR) methods. Both can be associated with different contrasts including IW contrast. The STIR method has the advantage of being more robust to field inhomogeneities, while CHESS benefits from increased signal-to-noise ratios. Technological progress in recent years has led to more homogenous magnetic fields, which in turn have favored a shift towards the increasing use of CHESS to benefit from more signal. More recently, it has become possible to use a Dixon-type readout in spin-echo-based sequences. Consequently, this fat suppression method gained interest in musculoskeletal imaging [50]. Indeed, it provides more homogeneous fat suppression than CHESS sequences, while providing better signal-to-noise ratios than STIR sequences [51–53]. Therefore, Dixon fluid-sensitive FSE sequences have been proposed as an alternative for knee imaging, providing homogeneous fat suppression and both fat and non-fat-suppressed images in one acquisition [23–25]. However, there are drawbacks to the Dixon sequence that have limited its widespread use in joint imaging in clinical practice. First, Dixon sequences require longer acquisition times in comparison to CHESS and STIR, a major downside for some applications, including joint imaging where spatial resolution cannot be compromised (Fig. 8). Second, protocols usually include non-fat-suppressed and fat-suppressed images with different TEs (associating a more sensitive sequence with a shorter TE and a more specific sequence with a longer TE), and there is less interest than for other applications such as the spine in having the same type of contrast repeated with and without fat suppression as provided by the Dixon method. Finally, other advantages of the Dixon technique, namely the ability to assess fat qualitatively and quantitatively, are not of primary interest for the imaging of joints. For these reasons, Dixon sequences are not typically used in the authors’ institution for joint imaging, while they are commonly employed for imaging of the extremities, bone marrow, musculoskeletal tumors, and degenerative and inflammatory disorders of the axial skeleton, as well as for whole-body imaging [50, 54–57].

### 3D gradient-echo sequences

3D imaging has many potential advantages including isotropic resolution allowing reformats in any plane, which can be adapted to assess cartilage in different areas of the joint while decreasing the effect of partial volume averaging (Table 1). 3D imaging comes, however, with technical challenges. The first clinically available sequences were based on gradient-echo acquisitions, which allowed for clinically reasonable acquisition times [58].

**Fig. 9:**
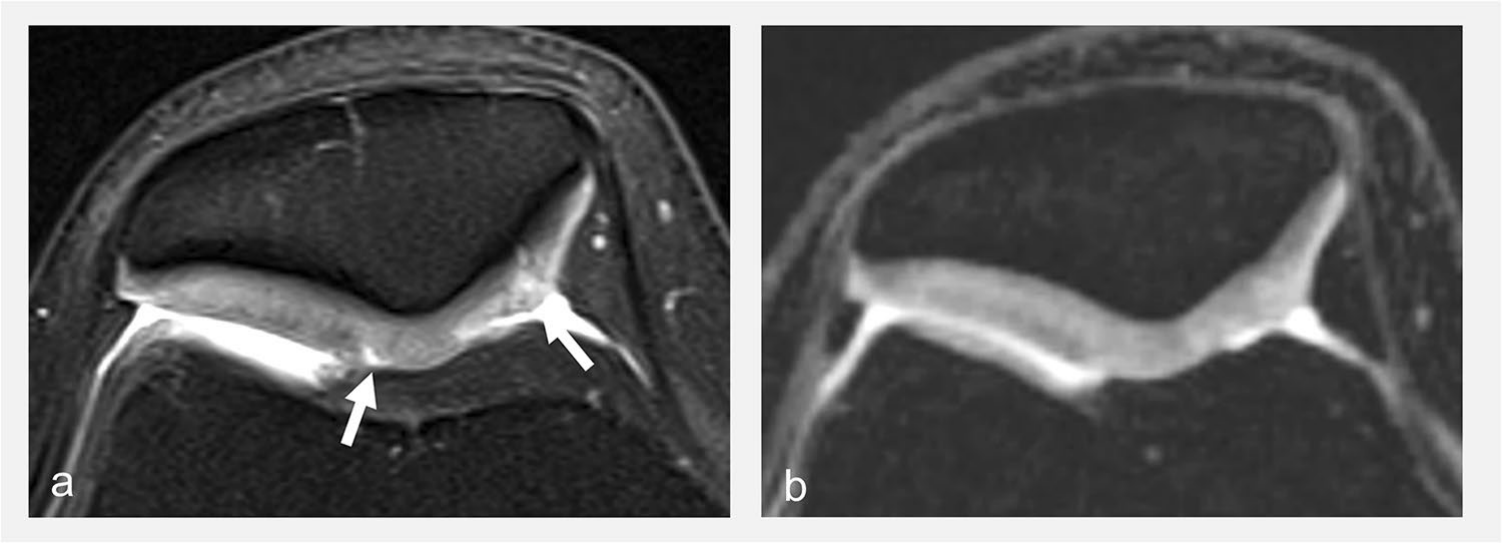
Transverse FS IW (**a**) and transverse reformat of 3D-double echo steady state (DESS) (**b**) MRI sequences obtained on a 3T MRI unit at the level of the patellofemoral joint. Cartilage fissures and intra-chondral high signal abnormalities are well visualized on FS IW image (arrows in **a**) but are occult on 3D-DESS

If the echo is generated using gradients instead of a refocusing pulse, then local inhomogeneities are not recovered and therefore the signal intensity is defined by the apparent transverse relaxation (T2*). In contrast, the main advantage of FSE sequences is that they use refocusing pulses to recover magnetization that is initially lost due to local field inhomogeneities [59]. Therefore, FSE sequences are less sensitive to inhomogeneities in the main magnetic field and the image intensities are influenced by the transverse magnetization (T2). A major advantage of gradient echo-based sequences is that the TR can be significantly shortened, which will typically result in fast sequences even when using a 3D excitation. In so doing, not only the longitudinal magnetization will not fully recover during that short period but also for very short TRs, there will be leftover transverse magnetization before the next excitation is applied. One way to deal with this remaining transverse magnetization is to spoil it, this fast low-angle shot (FLASH) technique results in rapid sequences [60], which were first used for the morphometry of cartilage in a research setting. If the transverse magnetization is not spoiled, then the magnetization will reach a steady state, producing image intensities mostly depending on the ratio T1/T2. A variant of this method is the Double Echo Steady State (DESS) sequence, which samples two echoes between two excitations. In the reconstruction, these echoes are combined to yield better signal-to-noise ratio (SNR) in the final image [61]. The improved SNR and the fact that cartilage presents a homogeneous signal and good contrast with surrounding tissues make 3D DESS sequences useful for cartilage morphometry and (semi-) automatic segmentation methods [62–64]. Such morphometric assessment is useful in the research setting but is not used in clinical routine yet. Furthermore, DESS sequences are limited by their long acquisition time and their suboptimal evaluation of cartilage pathology (Fig. 9) [65].

A variant of gradient-echo sequences is the volumetric interpolated breath-hold examination (VIBE) sequence [66]. The VIBE sequence was originally optimized for fast 3D fluid-sensitive acquisitions in the abdomen. The VIBE reconstruction uses partial-Fourier and sinc interpolation to achieve high-resolution images in short acquisition times. It has also shown value for the assessment of cartilage, including thinner cartilage, when associated with MR arthrography [67]. In our institution, these sequences are used in our MR arthrography protocol in combination with 2D FSE sequences.

Ultrashort echo time (UTE) sequences are a different variant of gradient-echo sequences allowing qualitative and quantitative assessment of short T2 structures with potential application for the assessment of osteoarthritis, including the detection of pathological processes in the deepest layer of cartilage and the detection of calcifications [68, 69]. Tissues with short T2 can be visualized by UTE sequences since the signal is sampled as soon as possible after the excitation, often trading higher image intensities for image artifacts such as blurring. A specific review will address these sequences in this issue [70].

### 3D turbo/fast spin echo fat-suppressed intermediate-weighted sequences

Gradient-echo-based sequences have some limitations. Image contrast shows significant variation depending on the parameters, leading to suboptimal sensitivity for the detection of cartilage, ligamentous and meniscal lesions [19, 41], together with a poor sensitivity to detect subchondral bone marrow edema-like signal [19, 71]. Therefore, over the last decades, efforts have focused on the development of 3D spin-echo-based sequences and have led to significant improvement in the performance of 3D imaging in assessing joint structures, currently showing similar diagnostic performance compared to 2D acquisitions [41]. The performance of 3D acquisitions is further increased at 3T and when using multiplanar reconstructions [41].

However, in clinical practice, 3D MRI has only gained limited popularity among radiologists and has not yet replaced 2D MRI in most standard imaging protocols [72]. The preference for 2D imaging can be partially attributed to two factors. Firstly, 2D sequences typically offer higher in-plane spatial resolution and better image contrast. On the other hand, 3D sequences require longer acquisition times, which makes them more susceptible to motion artifacts. If patient motion occurs during the acquisition, repeating the sequence can be problematic due to the longer acquisition (Table 1). In addition, 3D sequences show stronger blurring, and image contrast retains some T1 influence due to the long variable-flip-angle trains used to prevent prohibitive acquisition times.

### Synthetic MRI

Synthetic imaging has been defined as “additional contrasts that are derived from one or more sequences without requiring additional acquisition time” [73]. Specifically synthetic contrasts that can be tailored for specific applications, such as the morphological assessment of osteoarthritis, could be used to add useful additional image contrasts. Alternatively, synthetic contrasts could be used to reduce the duration of the exam by replacing actual acquisitions with synthetic contrasts.

One example is synthetic images generated from quantitative MRI (qMRI) maps [74, 75]. qMRI aims to accurately estimate tissue properties, such as T1 and T2 relaxation of the tissue. The results are typically represented in maps, i.e., images whose intensity corresponds to a physical unit (e.g., millisecond for relaxation). Although the primary use of these maps would be to derive biomarkers for early disease detection or treatment follow-up, they can also be used to synthesize conventional weighted images for the morphological assessment of cartilage. To that end, the quantitative maps are used in a physics model or simulation to generate an image that has the same appearance as a conventionally acquired image. The advantage of this approach is that one can simulate multiple synthetic images with different sequence properties (e.g., different echo times, providing image contrasts with different sensitivities/specificities tailored to the T2 values of the tissues to be examined), while also providing quantitative maps. Therefore, a single T2 mapping sequence can replace multiple T2-weighted acquisitions and thus shorten the overall acquisition time [2] (Fig. 3). Although still under evaluation, the field of synthetic contrasts shows promising results that may be helpful in future clinical routine. Comprehensive studies that include a diverse set of large patient cohorts are still required before such methods can be included in routine practice. Furthermore, some of these methods use AI, which should be used carefully in its appropriate context [76].

## Trends in technical development

### Faster imaging

Throughout the history of MRI, long acquisition times have always been a challenge which has been a focus of continuous research efforts. Over the past decades, various breakthroughs were made, and new methods are on the horizon showing that clinical protocols can be even further shortened.

One of the most established acceleration techniques is parallel imaging [77, 78]. In short, parallel imaging exploits the redundancy generated using multi-channel surface coils for receiving the MR signal. The image reconstruction uses a model of linear dependencies of neighboring k-space points to recover samples that were not acquired, hereby shortening the acquisition time. Typically, acceleration factors of two are possible, while maintaining the diagnostic performance [79]. However, parallel imaging results in non-uniform noise amplifications in the image.

For 2D imaging, simultaneous multi-slice (SMS) can be used to further accelerate the acquisition [80]. The sequence is modified to excite several slices at a time, resulting in superimposed MR signals from the different slices. The different signals can be separated by using similar reconstruction techniques as in parallel imaging, yielding an additional two-fold acceleration. It has been shown that a five-minute knee exam with five sequences can be achieved using this technique in combination with parallel imaging [81]. The disadvantage of the method is that it introduces additional noise to the image.

For 3D imaging, compressed sensing (CS) is a well-established method in the research setting and has become clinically available on various platforms in recent years [82]. In addition to parallel imaging, CS exploits the compressibility of MR images, i.e. the information contained in an MR image can be described by a few coefficients. This knowledge is used to recover missing samples in an iterative image reconstruction and allows for acceleration factors of four to five. Contrary to SMS, CS cannot be easily combined with traditional parallel imaging methods since both have contradicting requirements for the sequence-sampling scheme. While CS can also be applied to 2D imaging, it only can outperform traditional parallel imaging techniques for the most compressible types of images, i.e. high-resolution, isotropic 3D images. However, due to the nature of the image reconstruction, CS may introduce blurring, which can affect radiological reading.

**Fig. 10:**
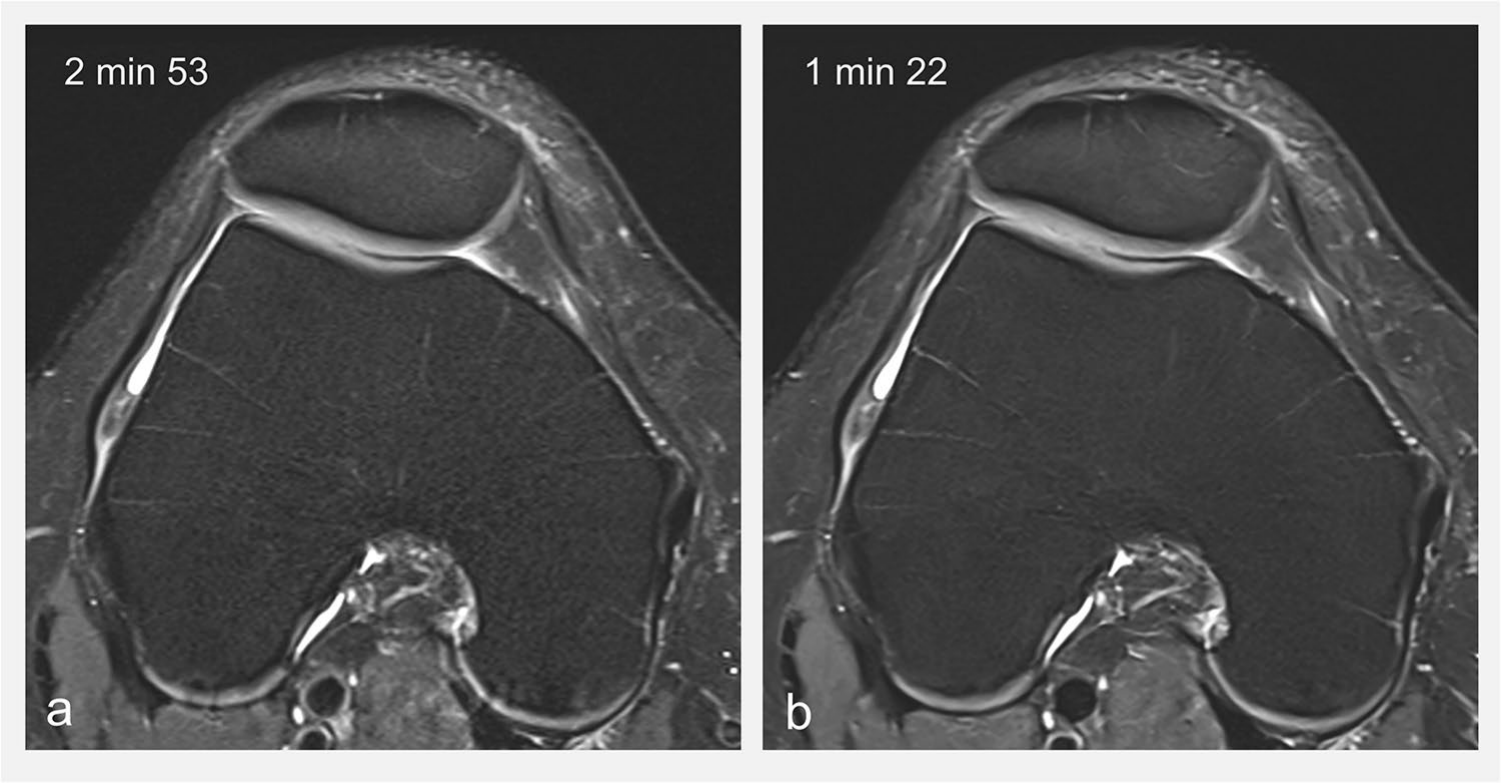
**a** FSE FS IW transverse image of the knee using the regular imaging protocol and **b** using AI acceleration that enables a reduction in acquisition time, while reducing image noise and increasing spatial resolution (in plane resolution increased from 0.37x0.52 mm^2^ in **a** to 0.24x0.32 mm^2^ in **b**)

The new acceleration methods currently in the pipeline are focusing on the use of AI. Specifically, most techniques are using deep convolutional neuronal networks trained to remove the artifacts in the image originating from under-sampled (i.e., accelerated) acquisitions. Researchers have achieved acceleration factors from four to eight in 2D knee acquisitions [83, 84]. While image quality appears to be high even with short acquisition times (Fig. 10), its potential is still being evaluated and validation is ongoing before translation to clinical routine. One downside of such approaches is that large datasets are required to train the AI. On one hand, it can be challenging to obtain these datasets due to additional efforts of data curation but also due to patient privacy concerns. On the other hand, it is still unknown how well these methods are generalizable, e.g., can an AI algorithm that was trained on one type of contrast be used on another type of contrast. Both concerns, data requirements and generalizability, are currently addressed in the research community, e.g. by using self-supervised methods [85].

### Unusual field strengths

Currently, typical clinical scanners have a field strength of 1.5 or 3 Tesla. However, over the past decades, scanners with different field strengths were approved for clinical use.

In 2017 the first 7 Tesla scanner was approved for clinical routine use. The improved SNR when using such an ultra-high field scanner can be directly used to improve the spatial resolution. Hence, improved imaging of small cartilage lesions or bone trabecular microstructure is expected. Nonetheless, research comparing different field strengths is still ongoing and early results show that only specific cases will clinically benefit from ultra-high field in comparison to a 1.5 or 3 Tesla exam [86]. Furthermore, the increased field strength comes with technical challenges such as field inhomogeneities and specific absorption rate limitations. These challenges may be overcome in the future using techniques such as parallel transmit radio frequency pulses [87].

On the other side of the field strength spectrum, modern low-field (e.g., 0.55 Tesla) scanners have recently become increasingly investigated and made commercially available in 2021. While the lower field strength results in reduced SNR and thus lower resolution, low-field systems are more affordable than higher field strengths and could enable broad use of MRI even in low-income countries. From a technical point of view, field inhomogeneities are less severe and artifacts introduced by metal implants are severely mitigated. Deep learning reconstruction algorithms hold great potential to reduce image noise, improve image quality, and thereby alleviate the long acquisition time. Ongoing research will show if the reduced image quality due to the lower field strength influences clinical decisions [88].

## Conclusion

For several decades, 2D FSE FS IW sequences have stood the test of time and have remained the keystone for the morphological evaluation of cartilage and of the joint in general. These sequences are routinely employed in multiple planes and commonly combined with other sequences, such as T1 and T2w sequences, which provide complementary information. While technological advancements have improved signal-to-noise ratio, fat suppression homogeneity, and spatial resolution, the image contrast provided by FS IW sequences remains the reference standard. Current research efforts aim to enhance these sequences by transitioning from 2D to 3D acquisitions, reducing examination times, and exploring other magnetic field strengths. Synthetic MRI has opened new possibilities for imaging with the simultaneous use of multiple image contrasts.

## Abbreviations

AI: Artificial intelligence
CHESS: Chemical shift selective saturation
CS: Compressed sensing
DESS: Dual echo steady state
FSE: Fast spin echo
FS: Fat-suppressed
IW: Intermediate-weighted
OA: Osteoarthritis
PDw: Proton density-weighted
T2w: T2-weighted
SMS: Simultaneous multi-slice
STIR: Short tau inversion recovery
UTE: Ultra-short TE

## References

[1] Roemer FW, Demehri S, Omoumi P, et al. State of the art: imaging of osteoarthritis-revisited. Radiology. 2020:2020192498. 10.1148/radiol.2020192498 32427556

[2] Roux M, Hilbert T, Hussami M, Becce F, Kober T, Omoumi P (2019). MRI T2 mapping of the knee providing synthetic morphologic images: comparison to conventional turbo spin-echo MRI. Radiology..

[3] Colotti R, Omoumi P, Bonanno G, Ledoux JB, van Heeswijk RB (2018). Isotropic three-dimensional T_2_ mapping of knee cartilage: Development and validation. J Magn Reson Imaging..

[4] Wirth W, Ladel C, Maschek S, Wisser A, Eckstein F, Roemer F. Quantitative measurement of cartilage morphology in osteoarthritis: current knowledge and future directions. Skeletal Radiol. 2022. 10.1007/s00256-022-04228-w PMC10509082 36380243

[5] Omoumi P, Mercier GA, Lecouvet F, Simoni P, Vande Berg BC (2009). CT arthrography, MR arthrography, PET, and scintigraphy in osteoarthritis. Radiol Clin North Am..

[6] Steinbach LS, Palmer WE, Schweitzer ME (2002). Special focus session. MR arthrography. Radiographics..

[7] White LM, Kramer J, Recht MP (2005). MR imaging evaluation of the postoperative knee: ligaments, menisci, and articular cartilage. Skeletal Radiol..

[8] Heuck A, Woertler K (2022). Posttreatment imaging of the knee: cruciate ligaments and menisci. Semin Musculoskelet Radiol..

[9] Potter HG, Linklater JM, Allen AA, Hannafin JA, Haas SB (1998). Magnetic resonance imaging of articular cartilage in the knee. An evaluation with use of fast-spin-echo imaging. J Bone Joint Surg Am..

[10] Bredella MA, Tirman PF, Peterfy CG (1999). Accuracy of T2-weighted fast spin-echo MR imaging with fat saturation in detecting cartilage defects in the knee: comparison with arthroscopy in 130 patients. AJR Am J Roentgenol..

[11] Link TM (2009). MR imaging in osteoarthritis: hardware, coils, and sequences. Radiol Clin North Am..

[12] Omoumi P, Teixeira P, Delgado G, Chung CB (2009). Imaging of lower limb cartilage. Top Magn Reson Imaging..

[13] Kijowski R, Davis KW, Woods MA (2009). Knee joint: comprehensive assessment with 3D isotropic resolution fast spin-echo MR imaging--diagnostic performance compared with that of conventional MR imaging at 3.0 T. Radiology..

[14] Rosas HG, De Smet AA (2009). Magnetic resonance imaging of the meniscus. Top Magn Reson Imaging..

[15] Nguyen JC, De Smet AA, Graf BK, Rosas HG (2014). MR imaging-based diagnosis and classification of meniscal tears. Radiographics..

[16] Peh WC, Chan JH (1998). The magic angle phenomenon in tendons: effect of varying the MR echo time. Br J Radiol..

[17] Bydder M, Rahal A, Fullerton GD, Bydder GM (2007). The magic angle effect: a source of artifact, determinant of image contrast, and technique for imaging. J Magn Reson Imaging..

[18] Pai A, Li X, Majumdar S (2008). A comparative study at 3 T of sequence dependence of T2 quantitation in the knee. Magn Reson Imaging..

[19] Jung JY, Yoon YC, Kim HR, Choe B-K, Wang JH, Jung JY (2013). Knee derangements: comparison of isotropic 3D fast spin-echo, isotropic 3D balanced fast field-echo, and conventional 2D fast spin-echo MR imaging. Radiology..

[20] De Smet AA (2012). How I diagnose meniscal tears on knee MRI. AJR Am J Roentgenol..

[21] Yoshioka H, Stevens K, Genovese M, Dillingham MF, Lang P (2004). Articular cartilage of knee: normal patterns at MR imaging that mimic disease in healthy subjects and patients with osteoarthritis. Radiology..

[22] Waldschmidt JG, Rilling RJ, Kajdacsy-Balla AA, Boynton MD, Erickson SJ (1997). In vitro and in vivo MR imaging of hyaline cartilage: zonal anatomy, imaging pitfalls, and pathologic conditions. Radiographics..

[23] Park HJ, Lee SY, Rho MH (2016). Usefulness of the fast spin-echo three-point Dixon (mDixon) image of the knee joint on 3.0-T MRI: comparison with conventional fast spin-echo T2 weighted image. Br J Radiol..

[24] Bastian-Jordan M, Dhupelia S, McMeniman M, Lanham M, Hislop-Jambrich J (2019). A quality audit of MRI knee exams with the implementation of a novel 2-point DIXON sequence. J Med Radiat Sci..

[25] Kammen BF, Padua EM, Karakas SP (2019). Clinical experience with two-point mDixon turbo spin echo as an alternative to conventional turbo spin echo for magnetic resonance imaging of the pediatric knee. Pediatr Radiol..

[26] Hunter DJ, Altman RD, Cicuttini F (2015). OARSI Clinical Trials recommendations: knee imaging in clinical trials in osteoarthritis. Osteoarthritis and Cartilage..

[27] Eckstein F, Winzheimer M, Hohe J, Englmeier KH, Reiser M (2001). Interindividual variability and correlation among morphological parameters of knee joint cartilage plates: analysis with three-dimensional MR imaging. Osteoarthritis Cartilage..

[28] Eckstein F, Yang M, Guermazi A, et al. Reference values and Z-scores for subregional femorotibial cartilage thickness - results from a large population-based sample (Framingham) and comparison with the non-exposed Osteoarthritis Initiative reference cohort. Osteoarthr Cartil. 2010;18(10):1275–83. 10.1016/j.joca.2010.07.010 PMC2982217 20691798

[29] Omoumi P, Michoux N, Larbi A (2017). Multirater agreement for grading the femoral and tibial cartilage surface lesions at CT arthrography and analysis of causes of disagreement. Eur J Radiol..

[30] Markhardt BK, Huang BK, Spiker AM, Chang EY. Interpretation of cartilage damage at routine clinical mri: how to match arthroscopic findings. Radiographics:2022220051. 10.1148/rg.220051 PMC9453290 35984752

[31] Vande Berg BC, Lecouvet FE, Maldague B, Malghem J (2004). MR appearance of cartilage defects of the knee: preliminary results of a spiral CT arthrography-guided analysis. Eur Radiol..

[32] Wissman RD, Ingalls J, Nepute J (2012). The trochlear cleft: the “black line” of the trochlear trough. Skeletal Radiol..

[33] Markhardt BK, Chang EY (2014). Hypointense signal lesions of the articular cartilage: a review of current concepts. Clin Imaging..

[34] Noyes FR, Stabler CL (1989). A system for grading articular cartilage lesions at arthroscopy. Am J Sports Med..

[35] Slattery C, Kweon CY (2018). Classifications in brief: outerbridge classification of chondral lesions. Clin Orthop Relat Res..

[36] Harris JD, Brophy RH, Jia G (2012). Sensitivity of magnetic resonance imaging for detection of patellofemoral articular cartilage defects. Arthroscopy..

[37] Devitt BM, Bell SW, Webster KE, Feller JA, Whitehead TS (2017). Surgical treatments of cartilage defects of the knee: Systematic review of randomised controlled trials. Knee..

[38] Hayashi D, Roemer FW, Link T (2022). Latest advancements in imaging techniques in OA. Therapeutic Advances in Musculoskeletal Disease..

[39] Hunter DJ, Guermazi A, Lo GH (2011). Evolution of semi-quantitative whole joint assessment of knee OA: MOAKS (MRI Osteoarthritis Knee Score). Osteoarthritis and Cartilage..

[40] Walter SS, Fritz B, Kijowski R, Fritz J. 2D versus 3D MRI of osteoarthritis in clinical practice and research. Skeletal Radiol. 2023. 10.1007/s00256-023-04309-4 36907953

[41] Shakoor D, Guermazi A, Kijowski R (2018). Diagnostic Performance of three-dimensional MRI for depicting cartilage defects in the knee: a meta-analysis. Radiology..

[42] Kijowski R, Blankenbaker DG, Davis KW, Shinki K, Kaplan LD, De Smet AA (2009). Comparison of 1.5- and 3.0-T MR imaging for evaluating the articular cartilage of the knee joint. Radiology..

[43] Omoumi P, Rubini A, Dubuc J-E, Vande Berg BC, Lecouvet FE (2014). Diagnostic performance of CT-arthrography and 1.5T MR-arthrography for the assessment of glenohumeral joint cartilage: a comparative study with arthroscopic correlation. Eur Radiol..

[44] Rubenstein JD, Li JG, Majumdar S, Henkelman RM (1997). Image resolution and signal-to-noise ratio requirements for MR imaging of degenerative cartilage. AJR Am J Roentgenol..

[45] Link TM, Majumdar S, Peterfy C (1998). High resolution MRI of small joints: impact of spatial resolution on diagnostic performance and SNR. Magn Reson Imaging..

[46] Pfirrmann CWA, Duc SR, Zanetti M, Dora C, Hodler J (2008). MR arthrography of acetabular cartilage delamination in femoroacetabular cam impingement. Radiology..

[47] Konstantinidis G, Mitchell M, Boyd G, Coady C, Ghosh S, Wong I (2021). Poor sensitivity of magnetic resonance arthrography to detect hip chondral delamination: a retrospective follow-up of 227 FAI-operated patients. Cartilage.

[48] Neumann J, Zhang AL, Bucknor M, et al. Acetabular cartilage delamination: performance of MRI using arthroscopy as the standard of reference. Acta Radiol. 2022:2841851221113966. 10.1177/02841851221113966 35903867

[49] Schmaranzer F, Lerch TD, Steppacher SD, Siebenrock KA, Schmaranzer E, Tannast M (2021). Femoral cartilage damage occurs at the zone of femoral head necrosis and can be accurately detected on traction MR arthrography of the hip in patients undergoing joint preserving hip surgery. J Hip Preserv Surg..

[50] Omoumi P (2022). The Dixon method in musculoskeletal MRI: from fat-sensitive to fat-specific imaging. Skeletal Radiol.

[51] Ma J, Singh SK, Kumar AJ, Leeds NE, Zhan J (2004). T2-weighted spine imaging with a fast three-point dixon technique: comparison with chemical shift selective fat suppression. J Magn Reson Imaging..

[52] Del Grande F, Santini F, Herzka DA (2014). Fat-suppression techniques for 3-T MR imaging of the musculoskeletal system. Radiographics..

[53] Kirchgesner T, Perlepe V, Michoux N, Larbi A, Vande BB (2017). Fat suppression at 2D MR imaging of the hands: Dixon method versus CHESS technique and STIR sequence. Eur J Radiol..

[54] Bacher S, Hajdu SD, Maeder Y, Dunet V, Hilbert T, Omoumi P (2021). Differentiation between benign and malignant vertebral compression fractures using qualitative and quantitative analysis of a single fast spin echo T2-weighted Dixon sequence. Eur Radiol..

[55] Zanchi F, Richard R, Hussami M, Monier A, Knebel JF, Omoumi P (2020). MRI of non-specific low back pain and/or lumbar radiculopathy: do we need T1 when using a sagittal T2-weighted Dixon sequence. Eur Radiol..

[56] Maeder Y, Dunet V, Richard R, Becce F, Omoumi P (2018). Bone marrow metastases: T2-weighted dixon spin-echo fat images can replace T1-weighted spin-echo images. Radiology..

[57] Chiabai O, Van Nieuwenhove S, Vekemans MC (2022). Whole-body MRI in oncology: can a single anatomic T2 Dixon sequence replace the combination of T1 and STIR sequences to detect skeletal metastasis and myeloma. Eur Radiol..

[58] Glaser C, D’Anastasi M, Theisen D (2015). Understanding 3D TSE sequences: advantages, disadvantages, and application in MSK imaging. Semin Musculoskelet Radiol..

[59] Hennig J, Nauerth A, Friedburg H (1986). RARE imaging: a fast imaging method for clinical MR. Magn Reson Med..

[60] Haase A, Frahm J, Matthaei D, Hänicke W, Merboldt KD (2011). FLASH imaging: rapid NMR imaging using low flip-angle pulses. J Magn Reson..

[61] Hargreaves BA (2012). Rapid gradient-echo imaging. J Magn Reson Imaging..

[62] Friedrich KM, Reiter G, Kaiser B (2010). High-resolution cartilage imaging of the knee at 3T: basic evaluation of modern isotropic 3D MR-sequences. Eur J Radiol..

[63] Bach Cuadra M, Favre J, Omoumi P (2020). Quantification in musculoskeletal imaging using computational analysis and machine learning: segmentation and radiomics. Semin Musculoskelet Radiol..

[64] Fujinaga Y, Yoshioka H, Sakai T, Sakai Y, Souza F, Lang P (2014). Quantitative measurement of femoral condyle cartilage in the knee by MRI: Validation study by multireaders. J Magn Reson Imaging..

[65] Schaefer FKW, Kurz B, Schaefer PJ (2007). Accuracy and precision in the detection of articular cartilage lesions using magnetic resonance imaging at 1.5 Tesla in an in vitro study with orthopedic and histopathologic correlation. Acta Radiol..

[66] Rofsky NM, Lee VS, Laub G (1999). Abdominal MR imaging with a volumetric interpolated breath-hold examination. Radiology..

[67] Vandevenne JE, Vanhoenacker F, Mahachie John JM, Gelin G, Parizel PM (2009). Fast MR arthrography using VIBE sequences to evaluate the rotator cuff. Skeletal Radiol..

[68] Bae WC, Dwek JR, Znamirowski R (2010). Ultrashort echo time MR imaging of osteochondral junction of the knee at 3 T: identification of anatomic structures contributing to signal intensity. Radiology..

[69] Omoumi P, Bae WC, Du J (2012). Meniscal calcifications: morphologic and quantitative evaluation by using 2D inversion-recovery ultrashort echo time and 3D ultrashort echo time 3.0-T MR imaging techniques--feasibility study. Radiology..

[70] Cheng KY, Moazamian D, Ma Y, et al. Clinical application of ultrashort echo time (UTE) and zero echo time (ZTE) magnetic resonance (MR) imaging in the evaluation of osteoarthritis. Skeletal Radiol. 2023. 10.1007/s00256-022-04269-1 PMC10323038 36607355

[71] Yoshioka H, Stevens K, Hargreaves BA (2004). Magnetic resonance imaging of articular cartilage of the knee: comparison between fat-suppressed three-dimensional SPGR imaging, fat-suppressed FSE imaging, and fat-suppressed three-dimensional DEFT imaging, and correlation with arthroscopy. J Magn Reson Imaging..

[72] Van Dyck P, Smekens C, Roelant E, Vande Vyvere T, Snoeckx A, De Smet E (2022). 3D CAIPIRINHA SPACE versus standard 2D TSE for routine knee MRI: a large-scale interchangeability study. Eur Radiol..

[73] Hilbert T, Omoumi P, Raudner M, Kober T (2023). Synthetic contrasts in musculoskeletal MRI: a review. Invest Radiol..

[74] Hilbert T, Sumpf TJ, Weiland E (2018). Accelerated T_2_ mapping combining parallel MRI and model-based reconstruction: GRAPPATINI. J Magn Reson Imaging..

[75] Warntjes JB, Leinhard OD, West J, Lundberg P (2008). Rapid magnetic resonance quantification on the brain: Optimization for clinical usage. Magn Reson Med..

[76] Omoumi P, Ducarouge A, Tournier A (2021). To buy or not to buy-evaluating commercial AI solutions in radiology (the ECLAIR guidelines). Eur Radiol..

[77] Griswold MA, Jakob PM, Heidemann RM (2002). Generalized autocalibrating partially parallel acquisitions (GRAPPA). Magn Reson Med..

[78] Pruessmann KP, Weiger M, Scheidegger MB, Boesiger P (1999). SENSE: sensitivity encoding for fast MRI. Magn Reson Med..

[79] Garwood ER, Recht MP, White LM (2017). Advanced imaging techniques in the knee: benefits and limitations of new rapid acquisition strategies for routine knee MRI. AJR Am J Roentgenol..

[80] Barth M, Breuer F, Koopmans PJ, Norris DG, Poser BA (2016). Simultaneous multislice (SMS) imaging techniques. Magn Reson Med..

[81] Del Grande F, Rashidi A, Luna R (2021). Five-minute five-sequence knee MRI using combined simultaneous multislice and parallel imaging acceleration: comparison with 10-minute parallel imaging knee MRI. Radiology..

[82] Lustig M, Donoho D, Pauly JM (2007). Sparse MRI: The application of compressed sensing for rapid MR imaging. Magn Reson Med..

[83] Knoll F, Murrell T, Sriram A (2020). Advancing machine learning for MR image reconstruction with an open competition: Overview of the 2019 fastMRI challenge. Magn Reson Med..

[84] Subhas N, Li H, Yang M (2020). Diagnostic interchangeability of deep convolutional neural networks reconstructed knee MR images: preliminary experience. Quantitative Imaging in Medicine and Surgery..

[85] Yu T, Hilbert T, Piredda GF et al. Validation and generalizability of self-supervised image reconstruction methods for undersampled MRI. arXiv preprint arXiv:220112535. 2022

[86] Pazahr S, Nanz D, Sutter R (2022). 7 T Musculoskeletal MRI: Fundamentals and Clinical Implementation. Invest Radiol..

[87] Padormo F, Beqiri A, Hajnal JV, Malik SJ (2016). Parallel transmission for ultrahigh-field imaging. NMR Biomed..

[88] Khodarahmi I, Keerthivasan MB, Brinkmann IM, Grodzki D, Fritz J (2022). Modern low-field MRI of the musculoskeletal system: practice considerations, opportunities, and challenges. Invest Radiol..

